# Total Facial Autologous Fat Grafting for Treating Skin Manifestations in Scleroderma

**DOI:** 10.3390/life12121997

**Published:** 2022-11-30

**Authors:** Ariel Berl, Ofir Shir-az, Noa Perk, Abraham Levy, Yair Levy, Avshalom Shalom

**Affiliations:** 1 Department of Plastic Surgery, Meir Medical Center, Kfar Saba 4428164, Israel; 2 Sackler Faculty of Medicine, Tel Aviv University, Tel Aviv 6997801, Israel; 3 Department of Internal Medicine E, Meir Medical Center, Kfar Saba 4428164, Israel

**Keywords:** scleroderma, systemic sclerosis (SSc), autologous fat grafting, adipose-derived stem cells

## Abstract

Systemic sclerosis (SSc) or scleroderma, is a rare, systemic autoimmune connective tissue disease that can cause fibrosis of cutaneous tissue and visceral organs. Facial involvement can have a deleterious effect on patients’ function, cosmetic appearance and quality of life. This study describes our experience and results with total facial autologous fat grafting for treating scleroderma. It includes 14 women and 3 men with SSc, at an average age of 51.3 years who underwent 32 autologous fat grafting surgeries between 2017–2022. The surgical technique is further described and demographic and surgical data, including preoperative and postoperative measurements were analyzed. Patients who had multiple surgeries ultimately received grafts with twice the volume of fat than in the first procedure. The oral opening increased an average of 33%. All patients reported improvement in quality of life and were very satisfied with the aesthetic outcomes. The use of autologous fat grafting to treat SSc patients successfully increased oral openings and improved facial manifestations. The procedure is reproducible, safe and leads to improvement in facial manifestations and patients’ quality of life. It can be repeated over time to preserve or enhance the results.

## 1. Introduction

Systemic sclerosis (SSc), also called scleroderma, is a rare systemic autoimmune connective tissue disease of unknown etiology. It is characterized by chronic inflammation that leads to fibrosis of cutaneous tissue and visceral organs. This process causes the skin to thicken due to deposits of collagen fibers, small vessel vasculopathy, and production of autoantibodies [1,2,3].

SSc is classified into two subtypes: diffuse scleroderma and limited scleroderma (localized). It is a relatively rare disease with a varying prevalence estimated to be 3–47/100,000, primarily affecting women of middle-age [4,5,6,7].

The pathophysiology of scleroderma is not fully understood; however, there are several hypotheses regarding disease development. A leading theory is that SSc is caused by a combination of several factors, including infectious, genetic, immune-mediated, traumatic, and iatrogenic. It is believed that the immune-mediated activation of T-cells causes a release of adhesion factors, pro-inflammatory and pro-fibrotic signals leading to excessive collagen production, and to a decrease in matrix metalloproteinases. In turn, this leads to collagen degradation [8].

The main pathogenic abnormality in the skin and internal organs is the over-activation of fibroblasts, which leads to secretion of elevated amounts of extracellular matrix and collagen. This in turn leads to progressive fibrosis of the skin and internal organs [9].

Facial involvement is reported in over 90% of patients. This has deleterious functional and cosmetic effects on patients’ lives, as it limits opening the mouth and facial expression, and creates a mask-like appearance [10]. One devastating presentation of the disease is microstomia, which is the result of sclerosis and thickening of perioral tissue. This results in major disability by affecting phonation, mastication, and oral hygiene [2,11]. A patient’s quality of life may be severely impaired by these disfiguring and debilitating changes [12].

Various methods have been used to validate and assess the degree of facial involvement and complications. Measuring the distance between the tips of the upper and lower incisors is the most common [10].

Several modalities for treating the manifestations of SSc on the facial skin and subcutaneous tissue have been described [13]. These include phototherapy [14], physical therapy [8], intense pulsed-light treatment [15], steroid injections [16], hyaluronidase injections [17,18] and surgical management [2,10,12,13].

Another modality for treating scleroderma is autologous fat grafting to the affected areas, which, has been described in several case series [19,20,21]. This method aids in filling defects but its major importance seems to be the transfer of adipose-derived stem cells (ASCs) which have regenerative potential [10,12,22]. The procedure of lipoaspiration and lipofilling is considered to have a favorable safety profile, is simple, and has been used for reversing fibrosis in various conditions such as scars, radio dermatitis, and localized forms of scleroderma [19,20,23]. ASCs have a great potential for use in several other therapeutic regenerative procedures [24,25].

This study describes our experience and results with total facial autologous fat grafting for the treatment of patients with scleroderma.

## 2. Materials and Methods

This retrospective study was conducted at Meir Medical Center from January 2017 to September 2022. Patients diagnosed with SSc who were referred by a rheumatologist specializing in the disease, to the Department of Plastic Surgery for surgical treatment, were eligible. All patients over the age of 18 years who consented for autologous fat grafting surgery were included.

Data abstracted from electronic medical records included oral opening before and a minimum of 3 months after surgery, donor sites for fat grafting, amount of lipoaspirate, volume of fat grafted, amount injected to each treatment area, and complications. All demographic and surgical data were stored on an Excel spreadsheet (Microsoft Corp., Redmond, WA, USA). Patients reported their satisfaction using a standard questionnaire, on a scale ranging from 1 (very poor) to 7 (very good).

Descriptive and univariate statistical analyses were performed using SPSS-21 (Statistical Package for the Social Sciences, IBM Corp., Armonk, NY, USA).

### 2.1. Ethics

All patients provided written informed consent for the surgical procedure. The study was approved by the Institutional Review Board and complied with the guidelines of the Declaration of Helsinki. Patients provided written authorization for use of images.

### 2.2. Surgical Technique

Following a detailed consultation regarding the intended procedure and signing an informed consent, surgical sites are marked, preoperatively. Most procedures are conducted under general anesthesia. Donor sites for autologous fat grafting include the abdomen, flanks and thighs. The entry point for infiltration is incised using an 11 blade and tumescent solution (composed of 1000 mL 0.9% normal saline and 1 mL 1:1000 adrenaline) is administered. After waiting 10 min, liposuction is commenced using a 3 or 4 mm multi-perforated cannula and is continued until sufficient fat is collected. Equalization of donor sites is achieved using a 4 mm basket cannula without suction. All aspirated fat is collected in a sterile container. Incision edges are approximated using Vicryl rapid 5/0 sutures (Ethicon, Somerville, NJ, USA) to prevent buildup of seroma. The collected aspirate in the container is left to sediment and excess fluid is removed. The adipose tissue collected is transferred to a 50 mL Luer- lok™ syringe and left to further sediment by gravity for 10 min. Excess oil and blood are removed and the remaining fat is transferred to 3 mL Luer- lok™ syringes. The fat is transferred between syringes via a stopcock valve ensuring that the macrografts are transformed to micrografts. When treating the tear troughs, we continue to process the fat to the size of nanografts and inject using a 1 mL syringe. The skin is incised with an 18G needle and fat is then injected to predetermined areas using Coleman™ micro infiltration cannulas. In most cases, we inject fat pan-facially.

The injected fat is gently massaged and dispersed to prevent deformities or sharp edges between treated and untreated areas. It is important to refrain from applying pressure on a single point to prevent visible deformities. When treating the perioral area, the endotracheal tube should be held firmly by the surgeon to prevent inadvertent movement of the tube. A finger should be placed inside the mouth behind the lips to guide fat grafting to the lips and to prevent puncture of the internal part of the lips.

**Figure 1:**
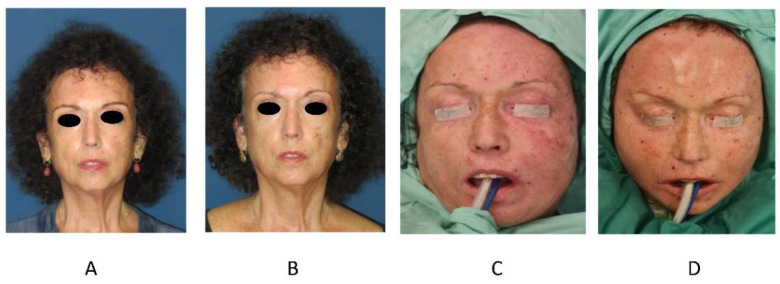
A 67-year-old female patient with SSc who underwent autologous fat grafting to the total facial area. A total volume of 110 cc was grafted from her flanks to her face. (**A**) Before, (**B**) 8 months after surgery, (**C**) During surgery, left side already grafted and right side before, (**D**) total face grafted.

After completing the procedure, the incisions are covered with Steristrips©. An abdominal binder or compression garment is worn at the donor sites for 6 weeks, preferably continuously during the first two weeks and for most of the day afterwards. Patients are discharged on the day of the procedure and given postoperative antibiotic coverage for one day. Follow-up visits are scheduled at 1 week, 1 month and 3 months postoperatively. (Figure 1).

## 3. Results

A total of 17 patients (14 women and 3 men) who underwent autologous fat grafting for the treatment of scleroderma were included in this study. Patients underwent a total of 32 procedures, the average per patient was 1.9 (range 1–4). The mean age during the procedure was 51.3 years (range 27–72). SSc subtype classification of patients included: 8 (47%) diffuse, 8 (47%) systemic and 1 limited (6%). The mean disease duration was 13.6 years (range 2–29).(Table 1) Medications used by patients presenting for surgery included iloprost (29%), mycophenolate mofetil (23%), tocilizumab (17%), and methotrexate (11%). Four patients were not receiving any immunosuppressive therapy. Serological markers included Scl-70 antibody (47%), rheumatoid factor (11%), anti-SSA antibody (11%), anti-SSB antibody (5%), and anti-centromere antibody (5%).

Facial areas treated included the forehead (n = 16, 50%), temporal area (n = 17, 53%), cheeks and zygomatic areas (n = 23, 72%), upper lip (n = 29, 90.6%), lower lip (n = 29, 90.6%), chin and mandibular areas (n = 16, 50%) and nose (n = 12, 37.5%). Surgical details are further described in Table 2.

Nine patients underwent multiple surgeries (5 had two surgeries, 2 had three surgeries and 2 had 4 surgeries). Surgeries were performed a minimum of 1 year apart.

When analyzing cases who underwent multiple procedures, the total fat volume injected to the face per surgery was increased by 2.01 times from the first to the last procedure. Donor areas included the flanks (n = 17), abdomen (n = 14), thighs (n = 4) and medial knee (n = 1).

**Figure 2:**
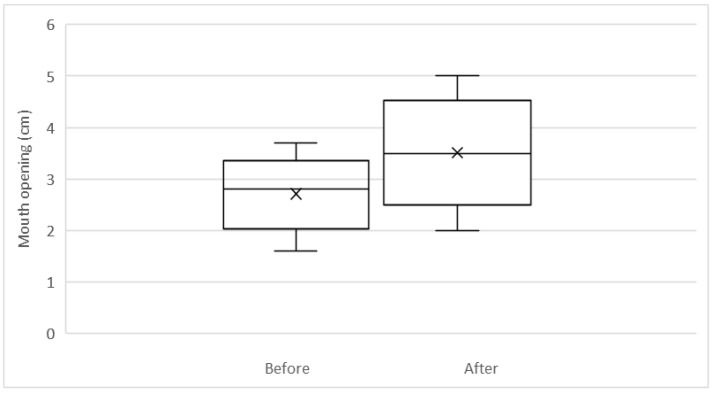
Measurements of oral opening (cm) before and after autologous fat grafting, preoperatively and at the 3-month postoperative follow up visit.

At the preoperative assessment, the mean opening between the upper and lower incisors was 2.8 cm (range 1.6–3.8 cm). Following the procedure, the oral opening improved by an absolute mean of 0.85 cm (range 0.2–2.5, *p* < 0.05, 95% CI 0.21–1.6). The average improvement from the preoperative measurement was 33% (Figure 2).

Three patients (17.65%) had a postoperative hematoma which resolved without intervention and 10 patients (58%) reported postoperative pain that subsided with the use of oral analgesics. No other major complications were documented on follow-up visits.

When queried regarding overall satisfaction (1–7 scale), patients reported a high satisfaction rate from the procedure (mean 5.2) and 88% would be willing to repeat it. The subjective satisfaction with functional improvement was 5.17 on average and the aesthetic outcome was 4.26. Postoperative pain was rated low and most patients (82%) reported that the procedure did not interfere with early return to their daily routines. Data reliability was tested by calculating the Cronbach’s Alpha, which was 0.773.

## 4. Discussion

Systemic sclerosis is a rare, autoimmune disease characterized by chronic inflammation, which leads to fibrosis of cutaneous tissue and visceral organs. Treatment and limiting the progression of the disease using therapeutic modalities is crucial for patients’ function and quality of life [1,2].

Various non-surgical modalities for treatment of sclerodermal manifestations on facial skin and subcutaneous tissue have been described [13]. Hyaluronic acid injections have been shown to be effective, yet the effect is limited due to material bioavailability [21]. The use of botulinum toxin has also been suggested to improve facial appearance by reducing muscle tension around the skin; however, this does not improve tissue atrophy [21]. Tewari et al. used phototherapy to treat microstomia in a patient with SSc. The protocol required 40 treatments and although the results were satisfactory, the major limitations were time to achieve the desired result and increased risk for developing skin cancer [14]. Intense pulse light for treatment of microstomia was described by Rosholm et al. Patients reported increased mobility, but a statistically significant difference in inter-incisal distance was not found [15]. Kumar et al. used intradermal injections of hyaluronidase with various steroid supplementations. The patient’s oral cavity opening improved but side-effects, including gastritis, occurred. [16]. Abbas et al. also used hyaluronidase injections into the mandible with improvement in oral aperture but there was a risk of severe discomfort at injection sites, angioedema and urticarial eruption [17].

The present study describes our technique using autologous fat grafting for the treatment of facial manifestations among patients with SSc. The results indicate that this method improves the aesthetic appearance and has a significant effect on tissue elasticity and pliability, along with an increase in the oral opening.

The results presented here show that among patients who underwent multiple procedures, we were able to inject twice the volume of fat into the treated areas between the first and last procedure. The ability to inject increasing amounts of fat over time, indicates that the treatment led to improved facial tissue elasticity.

Documented evidence shows that the improvements seen following autologous fat grafting are due to various mechanisms, including tissue regeneration [26,27]. The regenerative potential of ASC has been described and is believed to be the result of enhanced secretion of angiogenetic factors, along with immunomodulatory effects, which lead to decreased collagen deposition and increased elasticity [10,28,29]. The harvest of adipose tissue by liposuction yields a mixture of stromal vascular cells and adipocytes, both of which are important for the success of the procedure. The rich milieu of stromal cells and growth factors promotes improved angiogenesis and vasculogenesis; thereby, supporting graft survival. Fat grafts also provide structural support, which encourages stem cell proliferation and differentiation [2,30].

In the technique described here, collected fat is injected subcutaneously in all regions of the face. In areas such as the temporal region, we also inject to the superficial temporal fascia and superficial muscular aponeurotic system. The treatment and the introduction of cells with regenerative potential to all areas of the face, including supportive tissues and fibromuscular layers may be the main factors contributing to the increase in elasticity and functionality of all facial tissue observed. This increase in elasticity is believed to be the reason for the increased volume of grafted fat that can be injected in subsequent procedures.

Microstomia is a devastating result of facial involvement in patients with SSc that leads to disabilities in daily living and affects patients’ quality of life [2,12]. The use of autologous fat grafting for treating the perioral area is considered to have favorable outcomes [10,12,19,20,21,27,31,32].

Autologous fat grafting to treat fibrosis in various other conditions, such as scars, radio dermatitis, and localized forms of scleroderma has been reported to have good results, as well [19,20,23].

In the current study, patients treated in the perioral area experienced an improvement of 0.85 cm in the absolute mean oral opening. The average improvement from the preoperative measurement was 33%. As expected, the greatest improvement was noted in patients with severe microstomia. We do not expect to achieve a major improvement in the oral opening among patients with an inter-incisal aperture of 4 cm or more. Rather, we see increased tissue elasticity and slowing of disease progression.

To date, no technique for lipo-aspiration is considered superior to others [33]. In our technique, we leave the collected aspirate to sediment via gravity for at least 10 min prior to removing excess fluids. This technique has been used in other studies with satisfactory results [12,31].

Patient education prior to undergoing this surgery is of utmost importance. It should be emphasized that increasing improvement can be seen among patients who undergo several procedures. It is also important to describe the expected postoperative appearance of the face and early postoperative discomfort. The use of previous photographs of patients in the early postoperative period can be very helpful. These details must be presented to patients prior to surgery to ascertain that their expectations are reasonable and within the realm of expected results, and to help them undertake the decision-making process.

SSc patients are often treated by immunosuppressive drugs to halt disease progression. The process of wound healing is already a well described biological process with the inflammatory response a core part. Several of the immunosuppression medications taken by SSc patients have been linked to delayed wound healing and post-surgical complications [34]. Methotrexate is a widely used treatment for rheumatic diseases and has been associated with toxic effects on the skin and may impair wound closure. This effect has been widely studied, mainly in animal models [35]. Corticosteroids are also commonly used and have been linked to the disruption of the wound-healing process [36].

Autologous fat grafting surgery using the technique described above includes a minimum of skin punctures and incisions. Therefore, the effects of abnormal wound healing due to medications used for SSc patients are limited. Our cohort included patients treated with immunosuppressive medications; yet, no wound disruption was documented.

In our experience, the autologous fat grafting procedure has proven to be safe. Although some patients presented with hematomas (17.7%) and postoperative pain (58%), these side-effects are to be expected and can be managed conservatively.

Autologous fat grafting surgery can be performed under general anesthesia (as used here) or with local anesthesia supported by sedation and under the observation of a certified anesthesiologist. In patients presenting with severe microstomia, which may lead to a difficult intubation or in cases of respiratory or cardiac impairment due to disease progression, assessment by an experienced anesthesiologist is required to determine the safest anesthesia modality for the patient.

Fat grafting procedures are considered to be low-risk and are associated with low perioperative morbidity rates. Complications following fat grafting are rare but can include allergic reaction to the local anesthetic, hematoma formation, fat necrosis, oil cysts, perioperative bleeding, surgical site infections and fat embolism from injecting fat directly into a blood vessel [37]. Cerebral fat embolism following facial fat injection is a very rare and serious complication [37,38].

Scarring may also be considered a complication at both the donor and recipient sites. In the technique presented here, donor site scarring is minimal and hidden in body folds or below the bikini line. Recipient site scarring is usually invisible because needles are used to puncture the skin. Skin irregularities can occur in both the recipient and donor sites. The volume of fat grafted is usually low and therefore, irregularities at donor sites are minimal. Furthermore, the practices of crisscross aspiration and fat equalization assist in preventing this complication. In the recipient sites, fat is injected using fine Coleman™ cannulas and with 1–3 mL syringes; thereby, injecting small aliquots in a balanced fashion.

This study had a few limitations, including those related to its retrospective nature. This was a single center study and patient satisfaction was rated subjectively and not with a validated questionnaire. Another limitation was the inability to measure and calculate the survival of the grafted fat. The underlying disease process of patients with SSc might affect graft survival due to enhanced fibrosis and lack of stromal tissue to provide vascular support for the grafts. Accordingly, during injections, it is important not to over-stretch the skin, to prevent the risk of tissue necrosis. The number of ASCs varies between patients and donor sites and this might lead to varying results between procedures in the same patient and among different patients.

Additional large-scale studies are required to assess graft survival rates and to quantify the results of ASC grafts in this unique group of patients. Furthermore, studies stratified by disease pathophysiology and severity might shed light on the effects of these factors and on fat graft survival.

A major advantage of this study is the relatively large cohort composed of women and men and the relatively long follow-up period. Although facial and oral manifestations of SSC are more prevalent in women [1], our study included men who were affected by these features. The close collaboration with a rheumatologist experienced in treating SSc patients was a major asset and is imperative in the treatment of these patients. The multidisciplinary counseling assists in planning the timing of surgery, which is dictated by the therapeutic protocols and disease progression. This collaboration also allows patients to be treated multiple times over a period of years, which aids in tracking results and in improving the technique.

To date, treatment options for facial manifestations and microstomia related to scleroderma are limited. The various non-surgical modalities have limited efficacy and may have side-effects and complications.

## 5. Conclusions

The experience gained with using autologous fat grafting to treat patients with SSc indicates that this modality successfully increased the oral opening and improved facial manifestations. The procedure is reproducible, safe and leads to improvements in facial manifestations and in patients’ quality of life. Patients reported high subjective satisfaction and improvement in quality of life and were very satisfied with the aesthetic outcomes.

This procedure can be repeated over time to preserve or enhance results. Future large-scale research studies and implementation of a validated scale for assessing preoperative and postoperative results are warranted.

## Figures and Tables

**Table 1:** Baseline demographic characteristics.

Case	Sex	No. of Procedures	Age at First Procedure (Years)	SSc Subtype	Years since Diagnosis	BMI (kg/m^2^)
1	F	4	48	Diffuse	13	21.8
2	F	2	64	Diffuse	19	25.2
3	F	4	60	Diffuse	18	22.1
4	F	2	53	Diffuse	22	25.3
5	F	3	65	Diffuse	13	22.6
6	F	1	47	Limited	10	20
7	F	1	32	Diffuse	8	18.2
8	F	2	41	Systemic	29	20.4
9	F	2	51	Systemic	19	23.1
10	M	1	72	Systemic	8	24.2
11	F	1	61	Systemic	15	21.5
12	F	1	27	Diffuse	10	27.9
13	F	2	43	Systemic	1.5	21
14	M	1	64	Systemic	22	23.4
15	F	3	65	Systemic	7	29
16	F	1	38	Systemic	15	23.9
17	M	1	35	Diffuse	2	27.5

BMI, body mass index; SSc, systemic sclerosis.

**Table 2:** Autologous fat grafting surgical data.

Case	Procedure	Facial Area Treated								
		Forehead (n = 16)	Temporal Region (n = 17)	Zygoma/Cheek (n = 23)	Upper Lip (n = 29)	Lower Lip (n = 29)	Chin and Mandibular Areas (n = 16)	Nose (n = 12)	Total Volume/Procedure, mL (n = 32)	Donor Site
1	1				10	10			20	Abdomen
	2			10	7	8	10	3	38	Flank
	3		20	20	10	10	25	5	90	Hips
	4		20	20	10	10	20		80	Hips
2	1	2	10	30	5	5			52	Abdomen
	2	5	10	37	15	10	10		87	Abdomen
3	1	10		15	13	12	5	5	60	Hips
	2				12	15		3	30	Abdomen, hips
	3	10		15	15	15			55	Flank, abdomen
	4	15		20	20	20	10	5	95	Flank, knees
4	1	25	25	30	15	15	20	10	140	Abdomen
	2	20	20	20	10	10	10	10	100	Flank
5	1			10	13	14			37	Flank
	2				25				25	Flank
	3				10	13			23	Hips
6	1				15	8			23	Flank
7	1			24	6	15			40	Abdomen
8	1	20	10	20	4	4	2		60	Flank, abdomen
	2		20	50	14	10	20		114	Flank
9	1	20	20	44	10	12		6	112	Flank
	2	20	10	42	18	15	6	16	127	Abdomen
10	1				10	10	6	5	31	Abdomen
11	1	20	25	30			10	5	110	Abdomen
12	1								50	Abdomen
13	1		20	10	15	15			60	Flank
	2	5	10	10	5	5	15	2	52	Flank, abdomen
14	1	40		40	20	20			120	Flank, abdomen
15	1	20	20	30					110	Flank, abdomen
	2	10	46	54	10	10	22		152	Flank, abdomen
	3	30	30	90	10	10	10		180	Flank
16	1		8		8	7			21	Abdomen
17	1				10	10			20	Abdomen
Mean	1.7	17	19	29.17	11.42	11.17	12.56	6.25	72.31	--

## Data Availability

The data presented in this study are available on request from the corresponding author. The data are not publicly available due to privacy reasons.
